# Validating hospital admission criteria for decision support in pneumonia

**DOI:** 10.1186/1471-2466-14-149

**Published:** 2014-09-22

**Authors:** Barbara E Jones, Jason P Jones, Caroline G Vines, Nathan C Dean

**Affiliations:** Division of Pulmonary/Critical Care Medicine, University of Utah, 30N 1900E 701 Wintrobe, Salt Lake City, UT 84132 USA; Kaiser Permanente Southern California, Pasadena, CA USA; Department of Emergency Medicine, Intermountain Medical Center, Murray, UT USA; Division of Pulmonary/Critical Care Medicine, Intermountain Medical Center and the University of Utah, Salt Lake City, UT USA

**Keywords:** Pneumonia, Hospital admission decision, Hospital variation, Electronic decision support

## Abstract

**Background:**

We evaluated our previously derived admission criteria for agreement with physician decisions and outpatient failure among patients presenting to emergency departments (EDs) with pneumonia.

**Methods:**

Among patients presenting to seven Intermountain EDs in the urban region of Utah with pneumonia December 1 2009-December 1 2010, we measured hospital admission rates and outpatient failure, defined as either 7-day secondary hospitalization or death in 30 days for patients initially discharged home from the ED. We measured our admission criteria’s ability to predict hospital admission and its hypothetical rates of admission and outpatient failure with strict adherence to the criteria. We compared our admission criteria to other electronically calculable criteria, CURB-65 and A-DROP.

**Results:**

In 2,308 patients, admission rate was 57%, 30-day mortality 6.1%, 7-day secondary hospitalization 5.8%, and outpatient failure rate 6.4%. Our admission criteria predicted hospital admission with an AUC of 0.77, compared to 0.73 for CURB-65 ≥ 2 and 0.78 for A-DROP≥ 2. Hypothetical 100% concordance with our admission criteria decreased the hospitalization rate to 52% and reduced the outpatient failure rate to 3.9%, slightly better than A-DROP ≥ 2 (54% and 4.3%) and CURB-65 ≥ 2 (49% and 5.1%).

**Conclusions:**

Our admission criteria agreed acceptably with overall observed admission decisions for patients presenting to EDs with pneumonia, but may safely reduce hospital admission rates and increase recognition of patients at risk for outpatient failure compared to CURB-65 ≥ 2 or A-DROP ≥ 2.

## Background

Combined with influenza, pneumonia is the leading cause of death by infectious disease in the United States and costs $8-10 billion annually; the cost of inpatient care is up to 25 times that of outpatient care [1]. Studies show significant variation in the rates of hospitalization for pneumonia, both among hospitals [2] and individual physicians [3].

The selection of outpatient versus inpatient management is a major decision that should be guided by estimation of the patient’s illness severity [4]. Less ill patients are more satisfied with their care and return to daily activities faster if treated at home; [5–7] however, mortality may be greater for patients initially managed as outpatients but subsequently hospitalized [8]. A recent systematic review concluded that interventions to increase the proportion of patients treated in the community are safe and effective [9]. Implementation of electronic decision support that facilitates objective severity assessment at the site of care might also decrease the proportion of emergency department (ED) patients who fail outpatient treatment [10].

Several pneumonia severity assessment tools designed to estimate risk of 30-day mortality are calculable at the bedside from electronically available data (Table 1). Admission is recommended for patients with a CURB-65 score of 2 or greater [4]. The eCURB is an electronic version of CURB-65 that uses continuous, weighted variables and has demonstrated improved accuracy over CURB-65 [11]. The A-DROP score, proposed by the Japanese Respiratory Society [12], includes oxygenation in addition to CURB-65 features. The Japanese Respiratory Society recommends hospital admission for patients with an A-DROP score of 2 or greater. The IDSA-ATS 2007 minor criteria for severe community-acquired pneumonia (SCAP) includes oxygenation information, radiographic presence of multilobar infiltrates, platelet count, white blood cell count, and temperature. Fulfillment of 3 or greater SCAP criteria warrants admission to the intensive care unit (ICU) [4]. The Pneumonia Severity Index (PSI) includes comorbidities and has been found to be slightly more accurate at predicting mortality; [13] however, most current electronic health records lack accurate comorbidity data, making the PSI less feasible for electronic decision support.

**Table 1:** **Severity assessment tools and recommended hospital admission criteria for patients with pneumonia**

CURB-65:	Confusion (not oriented to person, place, or time)
Admission recommended for score of 2 or greater	Uremia: Blood urea nitrogen > 7 mmol/L or 9 mg/dL
	Respiratory Rate: ≥ 30 breaths per minute
	Blood Pressure: systolic < 90 mmHg or diastolic < 60 mmHg
	Age: > 65 years
A-DROP:	Age: ≥ 70 males, ≥ 75 females
Admission recommended for score of 2 or greater	Dehydration: blood urea nitrogen ≥ 210 mg/dL
	Respiratory failure: ambient SpO2 ≤ 90% or PaO2 ≤ 60 mmHg
	Orientation disturbance: confusion
	Pressure: systolic blood pressure ≤ 90 mmHg
SCAP:	*Major criteria:*
ICU admission recommended for ≥ 1 Major Criteria, OR ≥ 2 Minor Criteria	Invasive mechanical ventilation
	Septic Shock with the need for vasopressors
	*Minor criteria:*
	Respiratory rate ≥ 30 breaths/minute
	Multilobar involvement
	PaO2:FiO2 < 250 mmHg
	Confusion
	Uremia: Blood urea nitrogen ≥ 20 mg/dL
	Leukopenia: WBC count < 4000 cells/mm^3^
	Thrombocytopenia (platelet count < 100,000 cells/mm^3^
	Hypothermia (temperature ≤ 36C
	Hypotension requiring aggressive fluid resuscitation
Our admission criteria:	eCURB 30-day mortality estimate ≤ 5%
Admission recommended for any of the following criteria	PaO2:FiO2 ratio < 280 mmHg
	≥ 3 minor SCAP criteria
	Altitude adjusted PaO2:FiO2 ratio = Actual
	PaO2:FiO2/0.85

These tools accurately identify patients at low risk for 30-day mortality (although SCAP criteria are more accurate for identifying patients requiring ICU admission) [14]. However, their ability to predict other outcomes relevant to the admission decision, such as failure of outpatient therapy and subsequent hospitalization, have not been tested. Further, with the exception of eCURB, the criteria were developed using only populations of hospitalized patients with community-acquired pneumonia (CAP). Despite recommendations and availability, utilization of severity assessment tools has lagged behind use of other guideline elements [15, 16], due to difficulty in calculating scores at the bedside as well as environmental features that influence the admission decision. The ideal hospital admission criteria for electronic decision support would have the ability to extract information from the electronic health record to identify patients not only with low mortality risk, but also those at risk of failure of outpatient therapy, as measured by subsequent hospitalization or death.

We previously derived hospital admission criteria for an electronic decision support tool using a database of CAP patients from a single hospital that recommends admission for patients with any of: 1) eCURB 30-day mortality risk estimate of ≥5%, 2) ≥ 3 minor IDSA/ATS SCAP criteria; or 3) a PaO2:FiO2 ratio < 280 mmHg [3]. To validate these criteria and estimate their effects on hospitalization and outpatient failure, we gathered data for a new population of patients presenting to seven hospital EDs sharing a common electronic health record with both CAP and risk factors for healthcare-associated pneumonia (HCAP). We aimed to: 1) compare our admission criteria to A-DROP ≥ 2 and CURB-65 ≥ 2 for their agreement with actual hospital admission and potential to reduce hospital admissions and outpatient failures (secondary hospitalization or death); and 2) compare eCURB, CURB-65, and A-DROP for their ability to predict 30-day mortality for ED patients with CAP versus HCAP.

## Methods

### Setting

The study was performed at seven hospital EDs within the Intermountain Healthcare system in the urban regions of Utah, USA. Sizes range from a 58-bed community hospital with 4 critical care beds to a 440-bed university-affiliated tertiary care center with 84 critical care beds. Each hospital shares a common electronic health record used for documentation of patient encounters in the ED. The only available decision support for ED physician use during the study period was a paper guideline with CURB-65 scoring and antibiotic recommendations, but it was rarely utilized.

### Study subjects

**Figure 1:**
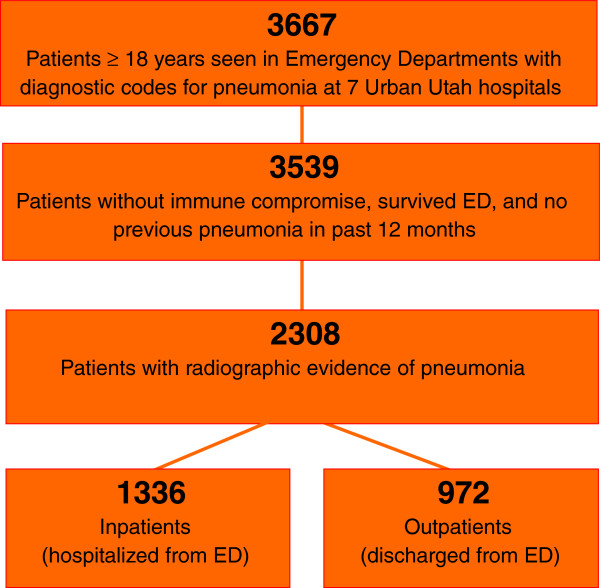
**Study population.**

Using a previously validated method [11], we identified all patients >18 years of age evaluated in the emergency department (ED) from December 1, 2009-December 1, 2010 with a primary diagnosis of pneumonia, defined by *International Statistical Classification of Disease, 9th edition* (ICD-9) codes 480–487.1, or secondary diagnosis of pneumonia and primary diagnosis of respiratory failure (518.x) or sepsis (785.52, 995.92, or 038.x). Patients diagnosed with aspiration pneumonia or immune-compromised conditions including AIDS or receipt of antiretroviral therapy, solid organ transplants, or hematologic malignancies were excluded. We included only the first episode of pneumonia in a given 12-month period. Chest radiograph and CT scan reports upon presentation to the ED were manually reviewed for radiographic evidence of pneumonia by three physicians, with an inter-rater reliability kappa of 0.83. Patients lacking radiographic evidence for pneumonia were excluded. Patients living in a skilled nursing facility, receiving long-term hemodialysis, or discharged from a hospital within the past 90 days were identified as patients with risk factors for HCAP. Figure 1 shows the study population.

### Measurements

We extracted data elements necessary to measure severity of illness with eCURB, CURB-65, A-DROP, and SCAP minor criteria (Table 1). Initial vital signs, orientation status, and laboratory results were obtained, based on previous work establishing that the initial measurements are more predictive of outcome than subsequent measurements [11, 14]. Values not found electronically were obtained by manual review of the ED record; this was required for about 10% of initial vital sign and oxygenation data. In 11% of patients (mostly outpatients), missing blood urea nitrogen levels were imputed with age-adjusted values, as previously described [12]. For patients without an arterial blood gas, we used the Ellis equation standardized with a temperature of 37.5C and a pH of 7.40 to estimate PaO2 [17]. Presence of multilobar infiltrates was determined from radiographic reports.

Using the electronic medical record, we identified hospital admission, length of stay, proportion of patients initially managed as outpatients and hospitalization within 7 days at any Intermountain Healthcare hospital (“7-day secondary hospitalization”) and proportion of patients initially managed as outpatients who died in 30 days (“30-day secondary death”). 30-day all-cause mortality was determined using the Utah Population Database [18]. The time frame of 7 days was chosen because most secondary admissions occurred within that period, and later admissions were mostly attributable to diagnoses other than pneumonia. Any patient with a 7-day secondary hospitalization or 30-day secondary death was defined as experiencing an “outpatient failure”.

### Analysis

Derivation of our admission criteria was previously described [3]. Our derivation resulted in two criteria: Admission Criteria 1 was unadjusted for altitude, and Admission Criteria 2,was adjusted for altitude by multiplying the PaO2:FiO2 ratio by 0.85, the ratio of barometric pressure at the altitude of the urban region of Utah (1400 m) to that of sea level.

The CURB-65, eCURB and A-DROP scores were tested for their ability to predict 30-day mortality using logistic regression and by calculating the area under the receiver operating characteristic curve (AUC). We also used the AUC to compare our admission criteria to CURB-65 ≥ 2 and A-DROP ≥ 2 for accuracy in predicting inpatient versus outpatient triage. AUC comparisons were tested for significance using the technique of DeLong [19]. We then calculated theoretical rates of hospital admission with 100% concordance of each admission criteria. Among patients discharged home from the ED, we determined the rates of outpatient failure when concordant with each admission recommendation versus outpatients triaged discordantly. Tests for significance were made with Chi-squared analyses.

All statistical analyses were performed using either the R statistical package [20] or Stata version 12.0 (Stata Corps, College Station, TX).

The study was conducted with approval by the Intermountain Healthcare Institutional Review Board and permission to access patient data was granted. As data were obtained retrospectively with no patient interaction, individual patient consent was not obtained.

## Results

We studied 2,308 pneumonia patients, 306 (13.3%) with risk factors for HCAP. The overall hospital admission rate was 58% (range 38-61% by hospital), 30-day mortality 6.1% (range 1.9-9.4%), 7-day secondary hospitalization rate 5.8%, and death within 30 days of outpatient triage 1.0%; the composite outpatient failure rate was 6.4%.

Table 2 demonstrates the expected rates of hospital admission and overall performance of our admission criteria (both adjusted for and unadjusted for altitude), CURB-65 ≥ 2, and A-DROP ≥2 for their ability to predict initial hospital admission. Strict concordance with any admission criteria would have reduced the overall hospitalization rate. The A-DROP ≥2 predicted hospital admission most accurately overall, with an AUC of 0.78. Our admission criteria unadjusted for altitude demonstrated better accuracy than the adjusted version (0.77 versus 0.73). All admission criteria predicted hospital admission less well for patients with risk factors for HCAP.

**Table 2:** **Actual versus expected rates of hospital admission for each admission criteria and AUC’s for ability of each criteria to predict hospital admission**

	All patients (N = 2,308)		CAP only (N = 2,002)		HCAP only (N = 306)	
	Hospital admission	AUC (CI’s)	Hospital admission	AUC (CI’s)	Hospital admission	AUC (CI’s)
Actual	58% (N = 1336)	NA	55% (N = 1099)	NA	77% (N = 237)	NA
Our admission criteria 1 (no altitude adjustment)	52% (N = 1192) p < 0.001	0.77 (0.76-0.79)	49%	0.78 (0.76-0.80)	69%	0.68 (0.62-0.75)
Our admission criteria 2 (altitude adjusted)	41% (N = 947) p < 0.001	0.74 0.73-0.76	38%	0.74 (0.72-0.76)	63%	0.67 (0.61-0.74)
CURB-65 ≥ 2	49% (N = 1130) p < 0.001	0.73 0.71-0.74	46%	0.73 (0.71-0.75)	65%	0.65 (0.58-0.71)
A-DROP ≥ 2	54% (N = 1244) p < 0.001	0.78 0.76-0.80	51%	0.79 (0.77-0.80)	71%	0.68 (0.61-0.74)

Results are reported for all patients, CAP patients, and HCAP patients.

Overall concordance with Admission Criteria 1 was 84% for a recommendation to hospitalize, and 70% for an outpatient recommendation. Table 3 demonstrates expected rates of outpatient failures with 100% concordance to each admission criteria. Patients triaged in concordance with any of the admission recommendations studied had lower outpatient failure rates. Of those patients discharged from the ED concordantly with Admission Criteria 1 (unadjusted for altitude), 3.9% had an outpatient failure, versus 16% of those discharged home from the ED discordantly with the criteria (p < 0.001). Outpatient failures were substantially higher for patients with HCAP than CAP (5.5% versus 17.4%, p < 0.001).

**Table 3:** **Actual versus expected rates of outpatient failure (7-day secondary hospitalization or 30-day outpatient death) for each admission criteria among rule-concordant versus rule-discordant outpatients**

	All patients			CAP only (N = 2,002)			HCAP only (N = 306)		
	Outpatient failure of concordant discharges	Outpatient failure of discordant discharges	p-value	Outpatient failure of concordant discharges	Outpatient failure of discordant discharges	p-value	Outpatient failure of concordant discharges	Outpatient failure of discordant discharges	p-value
Actual	6.4% (N = 62/972)			5.5% (N = 50/903)			17.4% (N = 12/69)		
Our admission criteria 1 (no altitude adjustment)	3.9% (N = 30/777)	16.4% (N = 32/195)	<0.001	3.8% (N = 28/736)	13.2% (N = 22/167)	<0.001	4.9% (N = 2/41)	35.7% (N = 10/28)	0.002
Our admission criteria 2 (altitude adjusted)	4.1% (N = 35/846)	21.4% (N = 27/126)	<0.001	4.1% (N = 33/802)	16.8% (N = 17/101)	<0.001	4.6% (N = 2/44)	40.0% (N = 10/25)	<0.001
CURB-65 ≥ 2	5.1% (N = 38/751)	10.9% (N = 24/221)	0.002	4.9% (N = 35/711)	7.8% (N = 15/192)	0.01	7.5% (N = 3/40)	31.0% (N = 9/29)	0.02
A-DROP ≥ 2	4.3% (N = 33/763)	13.9% (N = 29/209)	<0.001	10.6% (N = 31/724)	10.6% (N = 19/179)	0.001	5.1% (N = 2/39)	33.3% (N = 10/30)	0.003

Results are reported for all patients, CAP patients, and HCAP patients. CI = confidence interval.

Among the 30-day mortality predictors, eCURB was superior overall, with an area under the curve (AUC) of 0.83 versus 0.79 for A-DROP, and 0.78 for CURB-65 (p < 0.001). There was no statistically significant difference in performance between A-DROP and CURB-65 (p = 0.97). All mortality prediction tools predicted 30-day mortality less accurately for patients with HCAP: AUC for 30-day mortality for CAP and HCAP patients was 0.84 versus 0.74 for eCURB, 0.80 versus 0.69 for CURB-65, and 0.79 versus 0.74 for A-DROP (p < 0.001 for all). After adjustment with eCURB, patients with HCAP risk factors had an odds ratio for 30-day mortality of 3.0 (CI 2.25-4.0) compared to CAP patients.

## Discussion

Within a large population of patients presenting to 7 emergency departments with both healthcare-associated and community-acquired pneumonia, we found that all of the studied admission criteria demonstrated adequate agreement with actual admission decisions, with A-DROP ≥ 2 and our admission criteria unadjusted for altitude most closely aligned with physician decisions.

Complete concordance with our admission criteria would have reduced overall hospitalizations from 57% to 52%. Patients triaged in concordance with our admission criteria had a fourfold lower rate of outpatient failure than discordant triages, suggesting that tighter adherence to objective admission criteria could increase the number of patients managed safely as outpatients. As attempts to standardize the admission decision are undertaken, performing this type of analysis and establishing the optimal hospitalization rate and acceptable outpatient failure rates would likely increase adherence to recommendations.

Both A-DROP and our admission criteria consider oxygenation information, with a PaO2/FiO2 < 280 as a criterion for admission, while CURB-65 and eCURB do not. Previous care process models have added hypoxemia as a criterion for admission [20]. Our admission criteria unadjusted for altitude was more aligned with decision-making than using altitude adjustment, suggesting that physicians take into account hypoxemia as a disposition issue (need for home oxygen) rather than merely a measure of illness severity.

As previously found [11], the eCURB was superior overall to CURB-65 and A-DROP for 30-day mortality prediction, although all mortality predictors performed less well for patients with HCAP. Patients with HCAP in our study demonstrated higher rates of 30-day mortality, hospital admission and outpatient failure. All admission criteria performed more poorly in predicting admission for HCAP patients. This further supports that at least some of the proposed risk factors for HCAP confer a higher mortality risk compared to CAP. Clinicians have an appropriately lower threshold by hospital admission of these patients, regardless of the objective severity assessment. More work is needed to better define HCAP and to improve severity assessment tools and admission criteria for this patient population.

Overall concordance was higher with recommendations to hospitalize (84%) than recommendations to send home (70%), suggesting that additional factors likely contribute to a lower threshold for hospitalization in some patients. Previous studies attempting to identify reasons for non-adherence to objective admission recommendations found that presence of comorbid illness, risk of outpatient failure such as failure to take oral medications, and hypoxemia increases the hospitalization of low-risk patients [21]. Because components of any electronic severity assessment tool must be extracted electronically in real time and comorbidities are often missing in the current electronic health record, the severity assessment tools in this study all lack comorbid illness information. A prospective randomized control trial of a pneumonia care process model that used PSI plus hypoxemia as hospital admission criteria still demonstrated a 37% rate of hospitalization of low-risk patients by providers actively involved in the trial [20]. To identify reasons for non-adherence, the authors interviewed physicians that did not adhere to admission recommendations and found the presence of comorbid illness not taken into account by the PSI, a laboratory value, vital sign, or symptom that precluded ED discharge, or a recommendation from a primary care or consulting physician as the main reasons for low-risk admissions [22]. Social risk factors such as homelessness and mental illness have also been proposed as reasons for low-risk admissions [23–25]. As capabilities in our electronic health record evolve, future decision support will improve if it is able to integrate these factors with current severity assessment to provide recommendations.

There are several limitations to our study. Due to its observational design, we were only able to predict hypothetical impact on hospitalization rates and outpatient failure rates with strict adherence to the studied admission criteria. We recognize that the role of objective admission criteria is to enhance rather than replace physician judgment, and strict concordance with any objective admission criteria is neither a likely nor desirable effect of decision support. Our admission criteria was developed for optimal performance within the Intermountain system; however, the optimal hospital admission threshold likely varies appropriately across different settings and systems, based upon local patient populations, outpatient resources, and patient preferences. Studying hypothetical effects of different admission criteria on actual admission patterns, however, is useful in comparing the feasibility of their recommendations in a particular setting. We were unable to measure other meaningful outcomes such as time to return to work or patient satisfaction. Our design did not capture patients who were re-hospitalized outside the Intermountain system, and although the number of patient crossing over to other systems in our community is generally, this may have underestimates the true outpatient re-hospitalization rate. For those patients who were hospitalized against our criteria’s recommendation, we were unable to predict whether these patients would have had different outcomes were they cared for in an outpatient setting.

The true impact of our admission criteria can only be measured prospectively after implementation. However, this work validates the criteria and informs our expectations of the effects of our decision support tool on admission patterns and patient outcomes. Electronic decision support holds great promise in enhancing physician decisions for patients with pneumonia while preserving the ability to individualize care. More research is needed in the future to understand patterns in decision-making and to measure the effect of decision support implementation.

## Conclusion

Our admission criteria agreed acceptably with overall observed admission decisions for patients presenting to EDs with pneumonia, but may safely reduce hospital admission rates and increase recognition of patients at risk for outpatient failure compared to CURB-65 ≥ 2 or A-DROP ≥ 2.
